# *Staphylococcus aureus*: a model for bacterial cell biology and pathogenesis

**DOI:** 10.1128/jb.00106-25

**Published:** 2025-07-24

**Authors:** Mariana G. Pinho, Friedrich Götz, Andreas Peschel

**Affiliations:** 1 Instituto de Tecnologia Química e Biológica António Xavier, Universidade NOVA de Lisboa 98819 , Oeiras, Portugal; 2 Microbial Genetics, Interfaculty Institute of Microbiology and Infection Medicine Tübingen (IMIT), University of Tübingen 9188, Tübingen, Germany; 3 Excellence Cluster 2124 'Controlling Microbes to Fight Infections' (CMFI), University of Tübingen 9188, Tübingen, Germany; 4 Infection Biology, Interfaculty Institute of Microbiology and Infection Medicine Tübingen (IMIT), University of Tübingen 9188, Tübingen, Germany; 5 German Center for Infection Research (DZIF), partner site Tübingen, Tübingen, Germany; Geisel School of Medicine at Dartmouth, Hanover, New Hampshire, USA

**Keywords:** *Staphylococcus aureus*, model organism, bacterial cell biology, pathogenesis

## Abstract

*Staphylococcus aureus* has emerged as an important model organism in bacterial cell biology and pathogenesis due to its clinical relevance, genetic versatility, and adaptability. This review explores how *S. aureus* has contributed to advances in the fields of bacterial cell wall synthesis and cell division, particularly due to its minimal cell wall synthesis machinery and simple spherical shape. *S. aureus* has also been fundamental in antimicrobial resistance studies, particularly due to the increasing threat of antibiotic-resistant *S. aureus* strains. Furthermore, *S. aureus*’ dual lifestyle as both a commensal and a pathogen has provided key insights into host–microbe interactions, biofilm formation, and immune evasion strategies. This review underscores the importance of continued research on *S. aureus* as a basis for the development of novel antimicrobial strategies and vaccine approaches.

## INTRODUCTION

When talking about *Staphylococcus aureus*, one usually has in mind the diseases that can be caused by this bacterium, ranging from mild skin and soft tissue infections, such as abscesses and furuncles, to serious infections such as bloodstream infections, pneumonia, or bone and joint infections (1–6). Indirectly, staphylococci were already mentioned more than 2000 years ago by Jewish, Egyptian, Greek, and Roman historians, who described the symptoms of diseases typically caused by *S. aureus*. The Italian astronomer, humanist, poet, and philosopher Hieronymus Fracastorius (1477–1553) was an extremely far-sighted physician. Contrary to the prevailing doctrine, he theorized that certain infectious diseases could be transmitted by direct contact, at a distance, or via contaminated objects. In his work ‘*de contagione et contagiosis morbis et eorum curatione*’ (1546), he illustrated for the first time the theory of transmission of infectious diseases, such as syphilis, typhoid, plague, tuberculosis, rabies, leprosy, and skin diseases, by certain germs. It is likely that many of the skin diseases he described were caused by *S. aureus*. The medical historian Heinrich Haeser later recognized Fracastorius as the founder of epidemiography, the scientific description of epidemic diseases (7).

The name *Staphylococcus* (from *staphyle*, meaning “bunch of grapes”) was originally introduced by Ogston in 1883 to describe a group of micrococci causing inflammation and suppuration (8). The genera *Staphylococcus*, *Micrococcus*, and *Planococcus* were later grouped together within the family *Micrococcaceae* (9, 10). The grouping of these three genera into one family was based on three shared characteristics: they were Gram-positive, catalase-positive, and coccoid. This categorization did not reflect genetic relationships between those genera, but rather similarities based on morphology, habitats, and other shared physiological traits. However, the distinction between staphylococci and micrococci is particularly important, as some staphylococcal species are pathogenic, whereas micrococci are normally harmless saprophytes. Micrococci are normal commensal inhabitants of the human body and may play a vital role in maintaining the balance of the skin’s microbial flora. Although rarely associated with disease, they can occasionally cause infections in immunocompromised individuals (11). Facultative anaerobic cocci were classified in the genus *Staphylococcus*, while the more aerobic cocci were placed in the genus *Micrococcus* (12). As facultative anaerobes, *Staphylococcus* spp. are capable of growth under both aerobic and anaerobic conditions, enabling colonization of both oxic and anoxic regions of the human body. With the advancement of molecular biology, a clear distinction between staphylococci and micrococci was made based on DNA sequence composition (13). Members of the genus *Staphylococcus* have a DNA G + C content of 33–40%, whereas members of the genus *Micrococcus* have a high G + C content of 60–70%. DNA-DNA hybridization and DNA-rRNA hybridization studies (14, 15), along with comparative oligonucleotide cataloguing of 16S rRNA, clearly demonstrated the genetic difference between the genera *Staphylococcus* and *Micrococcus* (16). For the classification of staphylococci and micrococci according to modern criteria, see also Götz et al. (17).

As early as 1884, the genus *Staphylococcus* was divided into the two species: *Staphylococcus aureus* and *Staphylococcus albus* (18). This distinction was initially based on pigmentation, as most *S. aureus* strains produce characteristic golden-yellow pigments, whereas *S. albus*, later renamed *Staphylococcus epidermidis*, is mostly unpigmented and forms white colonies. However, pigmentation is an unstable marker, and many non-*S*. *aureus* species are also pigmented. Today, we know that the golden pigment is staphyloxanthin (19, 20), which protects *S. aureus* from oxidative stress (21), and the yellow pigment is 4,4'-diaponeurosporene, a precursor of staphyloxanthin (22). The NMR structure and biosynthetic pathway of staphyloxanthin were described in 2005 (20).

To date, 45 staphylococcal species and 24 subspecies have been described, using molecular methods. But how can *S. aureus* be distinguished from other staphylococcal species? Due to its high pathogenic potential, pathogenicity factors have primarily been used for differentiation. As early as 1903, Loeb categorized the genus *Staphylococcus* into two groups: coagulase-positive and coagulase-negative (CoNS) staphylococci. The coagulase test is based on the ability to produce free and/or cell wall-bound coagulase (clumping factor). While this classification remains relevant today, it is not sufficient to clearly identify *S. aureus*, as other staphylococcal species can also produce coagulase, such as *Staphylococcus intermedius*, *Staphylococcus schleiferi* subsp. *coagulans*, *Staphylococcus hyicus*, *Staphylococcus lutrae*, *Staphylococcus delphini*, and *Staphylococcus pseudintermedius* (23). However, these species are primarily associated with animals. For unequivocal identification of *S. aureus*, 16S rDNA sequencing and coagulase tests are required.

At the species level, the genetic diversity of *S. aureus* is relatively high. Evolution within the host is driven by genetic variation within its small genome (2.8–3.2 Mbp), which encodes 2,500–3,000 proteins and is carried on a single chromosome associated with one or more plasmids (24–27). The stable component (core genome) is complemented by a set of accessory genes (accessory genome) that can mediate antibiotic resistance, virulence, or immune evasion mechanisms and are often carried on mobile genetic elements. Antibiotic resistance genes are generally found on plasmids, transposons, or the staphylococcal cassette chromosome, whereas phages and pathogenicity islands carry virulence and immune evasion determinants (28).

The genetic diversity of *S. aureus* is also important in the laboratory setting, as different strains can yield varying results in the same assay or different phenotypes for the same mutation. This variability can lead to a lack of reproducibility between laboratories using different strains. Table 1 summarizes the most relevant *S. aureus* strains used in research, including their origins and main characteristics.

**TABLE 1:** Most relevant *S. aureus* strains used in laboratory studies

Strain	Origin	Characteristics	Research focus	Reference^*a*^
NCTC8325 (aka PS47 or RN1)	Isolated from a corneal ulcer in 1943	Reference strain initially studied by Richard Novick who used it for genetic studies, making it, and its derivatives, widely used laboratory strains, MSSA. The strain is defective in two regulatory proteins: RsbU (a positive activator of alternative sigma factor SigB) and TacR (an activator of protein A transcription)	Historical strain with multiple derivatives used for genetic studies	(29)
NCTC8325-4 (aka RN450)	Derivative of NCTC8325	Derivative of NCTC8325 cured of its three prophages, MSSA	Used mostly for genetic studies of *S. aureus*	(29)
RN4220	Derivative of NCTC8325-4	Laboratory strain obtained by chemical mutagenesis of NCTC8325-4 and selection for transformation efficiency. This resulted in 121 SNPs and four large-scale deletions, including a mutation in the SauI type I restriction–modification system, making it restriction deficient, MSSA	Widely used as an intermediate strain in genetic manipulation of *S. aureus,* as it is an efficient recipient of foreign DNA, which can be subsequently transferred to other *S. aureus* strains	(30)^*b*^
HG001	Derivative of NCTC8325	*rsbU*-repaired NCTC8325, which became more pathogenic, MSSA	Alternative model strain for physiological and virulence studies	(31)
HG003	Derivative of NCTC8325	*rsbU-* and *tacR-* repaired NCTC8325, MSSA	Alternative model strain for physiological and virulence studies	(31)
SH1000	Derivative of NCTC8325-4	*rsbU*-repaired NCTC8325-4, MSSA	Frequently used as a laboratory strain in studies that require an intact SigmaB pathway	(32)
Newman (aka NCTC 8178)	Isolated from secondarily infected tubercular osteomyelitis in male patient, UK, early 1950s	Highly virulent strain, MSSA	Commonly used for studies on virulence factors, pathogenesis, host-pathogen interactions; used extensively in animal models	(33, 34)
COL	Isolated from the air of an operating theatre in a hospital in England in the early 1960s. Name is an abbreviation of Colindale (the Central Public Health Laboratory where it was first studied)	One of the earliest MRSA strains, highly and homogenously resistant to methicillin; penicillinase-negative	Model for studying the molecular basis of methicillin resistance and the biochemistry of cell wall synthesis	(26, 35)^*c*^
N315	Isolated from pharyngeal smear of a Japanese patient, 1982	MRSA strain with inducible PBP2A; one of the first *S. aureus* strains to be sequenced.	Model strain to study mechanisms of methicillin resistance	(27, 36)
Mu50	Isolated from surgical wound infection refractory to vancomycin therapy, in infant hospital patient, Japan, 1996	One of the first clinically reported strains with reduced vancomycin susceptibility, MRSA; one of the first *S. aureus* strains to be sequenced.	Model strain to study the mechanism of reduced susceptibility to vancomycin mediated by thickened cell wall	(27, 37)
MW2	Isolated from 16 month-old American-Indian child with fatal septicaemia and septic arthritis in North Dakota, USA, in 1998	USA400 isolate, CA-MRSA	Model for studying CA-MRSA genetics and pathogenesis	(38, 39)
USA300 FPR3757	Isolated from the wrist abscess of an HIV-positive man at the San Francisco General Hospital, 2003	Epidemic community-associated methicillin-resistant *S. aureus* (CA-MRSA) from USA300 lineage. First USA 300 strain to be sequenced.	Used to study CA-MRSA virulence and antibiotic resistance	(24)
USA300 LAC	Isolated from a skin and soft tissue infection in a detainee from the Los Angeles County Jail, 2002	Epidemic CA-MRSA isolate from USA300 lineage.	Used to study CA-MRSA virulence and antibiotic resistance	(40, 41)^*d*^
JE2	Derivative of strain USA300 LAC	Derivative of strain USA300 LAC cured from its two plasmids p01 and p03, MRSA	Parental strain used to generate the Nebraska Transposon Mutant Library (NTML) of transposon mutants in non-essential genes (42) and the Lisbon CRISPRi Mutant Library (LCML), which allows depletion of essential proteins encoded by 200 genes/operons (43). Currently widely used for multiple studies.	(42)

^*a*^ References given correspond to the original reference describing the strain and to the reference describing the sequencing of the genome (when available).

^*b*^ A description of the isolation of RN4220 has not been published. A culture of NCTC8325-4 was treated with nitrosoguanidine and transformed with an erythromycin resistance *E. coli* plasmid, with selection for erythromycin. One colony was obtained, which became RN4220 (personal communication from Richard Novick).

^*c*^ Reference (35) has been used since the early 70s as the reference for strain COL. The strain described in this paper, culture nº 9204, is thought to be strain COL (personal communication from Brian Wilkinson).

^*d*^ Description of the isolation of USA 300 LAC can be found in reference (42).

The ability to genetically manipulate an organism is essential for its utility as a model organism. For decades, *S. aureus* was challenging to manipulate, primarily due to its restriction–modification systems, which prevented direct introduction of DNA from *Escherichia coli,* where cloning strategies are typically performed, into most laboratory or clinical strains of *S. aureus*. As a workaround, researchers commonly used the mutagenized and restriction-deficient strain RN4220 (Table 1) as an intermediate. Plasmids extracted from *E. coli* can be initially introduced into RN4220 by electroporation and subsequently transferred into other more relevant *S. aureus* strains using phage transduction. However, this approach involves a lengthy workflow, including the preparation of phage lysates and subsequent transduction steps. The development of *E. coli* strains by Monk and colleagues (44, 45), which mimics the methylation profiles of different *S. aureus* clonal complexes, overcame this limitation, enabling direct transformation of major *S. aureus* lineages and simplifying their genetic manipulation. Currently, a wide array of molecular tools for *S. aureus* is available, including plasmids with different copy numbers, antibiotic resistance markers, and reporter genes (46, 47), as well as comprehensive toolboxes of genetic parts (48) and CRISPR-based systems (43, 49–52). Additionally, the availability of whole-genome libraries of mutants covering both non-essential (42) and essential (43) genes greatly facilitates research involving *S. aureus*.

Due to its high clinical relevance and availability of molecular tools, *S. aureus* has become a very popular model organism for a wide range of questions related to bacterial cell biology and pathogenicity. In fact, among all bacterial species, *S. aureus* ranks second only to *E. coli* in the number of scientific publications (53). This review focuses on key research areas where *S. aureus* plays a particularly significant role as a model organism, namely, cell wall synthesis, antimicrobial resistance, cell division and morphogenesis, and microbe–host interactions. In addition, research on *S. aureus* has yielded seminal discoveries in the fields of metabolic adaptation, such as response to low abundance of CO_2_ (54), energy metabolism, explaining, for instance, how mutations in respiratory chain components lead to small-colony variants (55), or global regulatory networks, describing, for example, how *S. aureus* integrates multiple environmental and endogenous signals (56).

## *S. AUREUS* AS A MODEL TO STUDY CELL WALL SYNTHESIS

**Fig 1:**
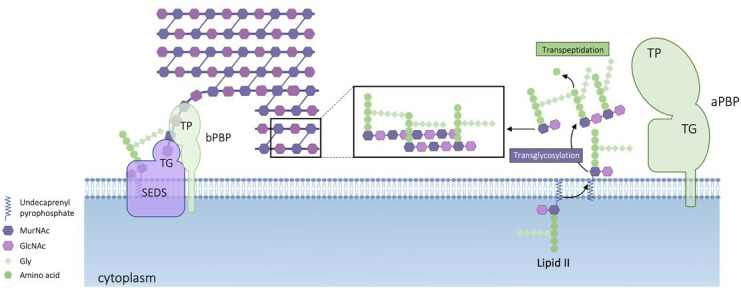
Schematic representation of the last steps of peptidoglycan synthesis in *S. aureus*. Peptidoglycan synthesis requires two reactions, transglycosylation and transpeptidation, which can be catalyzed either by a bifunctional class A PBP, namely, PBP2, or by a cognate pair consisting of a class B PBP with a transpeptidase (TP) domain and a SEDS (Shape, Elongation, Division, and Sporulation) protein with a transglycosylase (TG), also known as glycosyltransferase, domain. *S. aureus* has two of these pairs: PBP1/FtsW, dedicated to septum synthesis during cell division, and PBP3/RodA, involved in the mild elongation of *S. aureus* cells. PBP4, a monofunctional transpeptidase, is also present and is responsible for the high level of peptidoglycan crosslinking characteristic of *S. aureus*. MRSA strains have an additional PBP, PBP2A, a monofunctional transpeptidase that is thought to cooperate with the transglycosylase domain of PBP2, allowing peptidoglycan synthesis in the presence of otherwise inhibitory concentrations of β-lactam antibiotics.

The first studies of the *S. aureus* cell wall date back to the late 1950 s, driven by a strong interest in understanding the mode of action of antibiotics, particularly β-lactams, and their effects on this pathogen. Over time, *S. aureus* proved to be an excellent model organism for cell wall research, one of the reasons being its relatively low number of penicillin-binding proteins (PBPs) (57). PBPs are the primary enzymes responsible for synthesizing peptidoglycan, the main structural component of the bacterial cell wall. This polysaccharide is composed of glycan strands made of alternating residues of N-acetylglucosamine and N-acetylmuramic acid, crosslinked by short peptide bridges (Fig. 1). *S. aureus* has four PBPs, with methicillin-resistant *S. aureus* (MRSA) strains carrying the additional PBP2A (aka PBP2´), which confers resistance to β-lactam antibiotics (in comparison, *Bacillus subtilis* has 16 PBPs) (57, 58).

Among *S. aureus* PBPs, PBP2 is the only bifunctional protein, as it can synthesize glycan strands of peptidoglycan, through a transglycosylation reaction, and also crosslink these strands via peptide bridges, through a transpeptidation reaction (59, 60). Its activity takes place mostly, but not exclusively, at the septum (61). PBP1 and PBP3, on the other hand, are monofunctional transpeptidases that depend on the activity of an additional transglycosylase (also known as glycosyltransferase) for peptidoglycan synthesis (62, 63). This role is fulfilled by proteins from the SEDS (Shape, Elongation, Division, and Sporulation) family, whose function remained enigmatic for decades, until their activity in peptidoglycan polymerization was demonstrated by the groups of D. Rudner, T. Bernhardt, and J. Errington (64, 65). In *S. aureus*, the cognate partner of PBP1 is FtsW and that of PBP3 is RodA (66). PBP1/FtsW are septal peptidoglycan synthases that move around the division site to construct the division septum (66, 67), while PBP3/RodA act at the outer edge of the division septum contributing to the mild elongation observed in *S. aureus* cells (66). Lastly, PBP4, a low-molecular-weight monofunctional transpeptidase, is responsible for the extensive secondary crosslinking that is characteristic of *S. aureus* peptidoglycan (68, 69). The small number of *S. aureus* PBPs has allowed the understanding of the role of each of these proteins, a task that has proven difficult in other bacterial species.

While all *S. aureus* strains have the four native PBPs 1–4, only MRSA strains carry the additional PBP2A (70). This is a monofunctional transpeptidase with low affinity for β-lactams, enabling peptidoglycan synthesis even in the presence of otherwise inhibitory concentrations of antibiotics (70–72). PBP2A is thought to cooperate with the bifunctional PBP2, which provides glycosyltransferase activity in the presence of β-lactams (73).

Interestingly, the gene encoding PBP2A, *mecA*, was acquired from an extraspecies source, likely *Staphylococcus sciuri* (74, 75) or *Staphylococcus fleurettii* (76). However, the mere introduction of *mecA* into the genome of *S. aureus* is not sufficient to confer resistance to β-lactam antibiotics (77–82). This resistance requires additional adaptive changes involving multiple factors, making it challenging to determine the precise role of each contributor. The integration of PBP2A into *S. aureus* provides an interesting model for studying how a foreign protein becomes a functional part of a complex machinery, such as the one responsible for peptidoglycan synthesis.

The peptidoglycan mesh that protects bacteria is not only a target for β-lactam antibiotics (see below) but also a target for one of the first lines of defense used by the host innate immune system against invading pathogens—lysozyme. The bacteria-killing activity of this muramidase, which hydrolyzes the linkage between repeating glycan sugars resulting in bacterial lysis, was initially identified by Fleming in 1922 (83). To counteract the activity of lysozyme, some bacteria, including *S. aureus*, undergo O-acetylation of peptidoglycan, a modification that sterically hinders binding of lysozyme, rendering it inactive (84). The enzyme that confers lysozyme resistance to *S. aureus* is the peptidoglycan O-acetyltransferase (OatA) that O-acetylates N-acetylmuramic acid at the C6 position (85, 86). While O-acetyl groups in bacterial cell walls were initially identified in other bacteria, it was in *S. aureus* that their location in the N-acetylmuramic acid residues was determined (87).

Because *S. aureus* is resistant to lysozyme, laboratory procedures that require lysis through cell wall digestion typically use lysostaphin, an enzyme produced by *Staphylococcus simulans* and first described in 1964 (88). Lysostaphin is an endopeptidase that specifically targets the pentaglycine cross-bridges within the peptidoglycan of *S. aureus* and other staphylococcal species (89). In lysostaphin-producing *S. simulans*, the gene encoding lysostaphin (*lss*) is located on a plasmid that also carries the gene encoding the lysostaphin immunity factor (*lif*) (90). Interestingly, the Lif enzyme mediates resistance to lysostaphin by modifying the peptidoglycan structure through the incorporation of serine residues into the interpeptide bridges. This structural alteration prevents lysostaphin from effectively recognizing and cleaving the cross-bridges, exemplifying a canonical mechanism of producer-mediated self-protection.

Another important modification of peptidoglycan is the amidation of the D-glutamic acid present in the second position of the stem peptide, resulting in the formation of D-iso-glutamine (91). Although this modification was known since the 1960s, it took five decades to uncover the enzymes responsible for this amidation, through studies done in *S. aureus* (92, 93). Peptidoglycan amidation reduces the negative charge of the polymer, thereby decreasing bacterial susceptibility to innate immune defense mechanisms mediated by cationic molecules such as defensins (92). These studies confirm that *S. aureus* remains an important model organism for studying various aspects of peptidoglycan synthesis.

## *S. AUREUS* AS A MODEL TO STUDY CELL DIVISION

*S. aureus* has emerged as a valuable model for studying cell division, not only due to its clinical relevance but also because its spherical shape offers unique advantages for fluorescence microscopy, a critical tool for these studies. Traditional model organisms, like *E. coli* and *B. subtilis*, are rod-shaped, and their morphogenesis involves peptidoglycan incorporation at two distinct sites: the lateral wall (for cell elongation) and the division septum (94, 95). These processes are mediated by two specialized protein complexes, the elongasome and the divisome, both of which include, among other proteins, a cytoskeletal protein that organizes the complex and proteins required for peptidoglycan synthesis. The elongasome is organized by the actin-like protein MreB and coordinates the insertion of peptidoglycan into the lateral wall, enabling cell elongation (96–98). In contrast, the divisome, orchestrated by the tubulin-like protein FtsZ, assembles at the midcell to drive membrane invagination and peptidoglycan synthesis at the division septum (99, 100).

*S. aureus* cells are nearly spherical and therefore lack a canonical MreB elongasome, although mild elongation has been reported (66). This simplified morphology, combined with the presence of a single cell wall synthesis machinery and a reduced number of PBPs (57), makes *S. aureus* an attractive model for studying cell division. Compared to organisms with more complex shapes, *S. aureus* offers lower redundancy due to the low number of proteins involved in cell wall synthesis and in morphogenesis (58), simplifying the study of these processes.

The spherical shape of *S. aureus* provides an additional advantage for microscopy studies of cell division. In rod-shaped cells, division typically occurs along a plane perpendicular to the longer axis of the cell (101). Therefore, when these cells are placed on a glass slide, their division plane is perpendicular to the imaging plane. In contrast, spherical *S. aureus* cells lie in random orientations on the slide, so the division plane is parallel to the imaging plane in a significant fraction of the population. This constitutes an important advantage because the resolution in microscopy is highest within the imaging plane (102).

The components of the divisome have been identified over the past few decades and are relatively conserved across bacteria, including *S. aureus* (58). However, understanding how the divisome is assembled and disassembled, or how it drives cytokinesis, has been more challenging. In 2017, two seminal studies demonstrated that FtsZ undergoes treadmilling, a dynamic behavior of cytoskeletal filaments, where they extend at one end while simultaneously shortening at the other, driven by the ongoing addition and removal of protein subunits (103, 104). This behavior was proposed to organize septal cell wall synthesis and to control the rate of septum synthesis in *B. subtilis* but not in *E. coli* (103, 104). The ability to visualize ongoing cytokinesis in live *S. aureus* cells at high resolution helped resolve this discrepancy by showing that cytokinesis occurs in two stages (105). The first is a slower stage in which FtsZ treadmilling is required, presumably to promote condensation of FtsZ filaments (105, 106). This is followed by a second, faster stage, during which FtsZ treadmilling becomes dispensable and peptidoglycan synthesis becomes the major driving force of cytokinesis (105). Consistent observations have since been made in *B. subtilis* cells dividing while confined standing upright, enabling observation of their divisome in a plane parallel to the slide (107, 108).

Besides significantly contributing to the understanding of the role of the central divisome protein, FtsZ, *S. aureus* also serves as a valuable model for studying other divisome components, particularly the subcomplex composed of PBP1/FtsW/DivIB/DivIC/FtsL. Within this subcomplex, PBP1/FtsW are septal peptidoglycan synthases that move around the division site powered by their synthase activity (67), and DivIB/DivIC/FtsL are regulators of division that act at different steps to ensure efficient morphogenesis (109–111).

**Fig 2:**
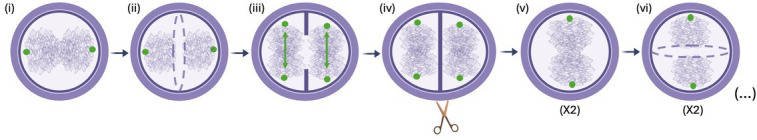
Geometry of cell division in *S. aureus*. (i) The cell cycle of *S. aureus* begins with a partially replicated chromosome, with newborn cells usually containing two origins of replication (green circles). (ii) The divisome (dashed purple line) assembles in the DNA-free plane between the two segregating chromosomes, leading to septum synthesis at this site. (iii) Chromosome segregation (green arrows) occurs along the long axis of each hemisphere of a mother cell. (iv) Once the septum is fully formed, peptidoglycan hydrolases split it, generating two daughter cells. (v, vi) As chromosome segregation is initiated in the mother cell, each daughter cell is born with a single plane (dashed line), where the next division can occur without bisecting the nucleoid. This plane is perpendicular to the previous septum. Therefore, chromosome segregation plays a key role in determining the division site in *S. aureus*.

The division site selection in *S. aureus* follows a different geometry compared to most well-studied organisms. While rods and ovococci divide using the same division plane, always perpendicular to the long axis of the cell, *S. aureus* divides along consecutive orthogonal division planes (112–114). In *S. aureus*, chromosome segregation occurs along the long axis of each hemisphere of a mother cell (115) (Fig. 2). This generates a DNA-free plane in the middle of the two segregating chromosomes, perpendicular to the previous septum, where the next division can occur without bisecting the nucleoid (112, 116). Therefore, chromosome segregation is the major determinant of division site placement in *S. aureus*, although other factors may also contribute to increasing accuracy (112, 117–119). The differences in mechanisms for division site placement between species with different shapes highlight the importance of studying diverse model organisms to understand the enormous diversity in bacteria, even in fundamental cellular processes such as cell division.

## *S. AUREUS* AS A MODEL TO STUDY ANTIBIOTIC RESISTANCE

When penicillin, a β-lactam that targets PBPs, became available in the 1940s, it was particularly helpful for the treatment of *S. aureus* infections. However, *S. aureus* was one of the first pathogens to develop penicillin resistance (120), and it became a model organism for the study of antibiotics’ mode of action, mechanisms of resistance, their horizontal transfer, and evolution (121). As a result, *S. aureus* is now one of the best studied representatives of the group of highly antibiotic-resistant bacterial pathogens named ESKAPE, after the first letter of each genus: *Enterococcus faecium/Enterococcus faecalis*, *S. aureus*, *Klebsiella pneumoniae*, *Acinetobacter baumannii*, *Pseudomonas aeruginosa*, and *Enterobacter* spp. (122).

The first reported *S. aureus* penicillin resistance mechanism was based on the extracellular penicillin-inactivating penicillinase (123). This enzyme became a paradigm for antibiotic-deactivating enzymes, members of which contribute to resistance to several other antibiotics. Penicillinase is usually encoded on plasmids, which are exchanged among pathogen clones, for instance, by transducing phages, thereby contributing to the fast dissemination of resistance (124). The S. *aureus* penicillinase was also one of the first described bacterial lipoproteins, bearing the typical thioester-linked lipid moiety at its N-terminus, thereby tethering the enzyme to the bacterial surface (125).

When penicillinase-insensitive β-lactams became available, the emergence of MRSA led to the discovery of the alternative PBP2A, which confers broad β-lactam resistance (70, 126). PBP2A is now a paradigm for an antibiotic resistance mechanism based on an altered, antibiotic-insensitive target protein. MRSA clones remain prevalent causes of *S. aureus* infections around the world with only limited options for effective treatment (127).

The most frequently used antibiotic for the treatment of MRSA infections is the glycopeptide vancomycin, which inhibits peptidoglycan synthesis by binding to the terminal D-Ala-D-Ala amino acids in the stem peptide of the peptidoglycan precursor, thereby preventing the access of PBPs to their substrate. Vancomycin-intermediate-resistant *S. aureus* (VISA) represents a problem of increasing concern because the VISA phenotype is difficult to diagnose, and there are only very limited alternative therapy options available (37, 128, 129). The VISA phenotype is based on multifactorial, complex changes in the structure of the cell wall, which involve increased cell wall thickness and decreased crosslinking (increasing the number of free D-Ala-D-Ala antibiotic targets) (130). The underlying regulatory mechanisms have helped to unravel important processes of cell wall synthesis and homeostasis (131).

Besides VISA, the emergence of highly vancomycin-resistant *S. aureus* (VRSA) strains became a major concern following the isolation of high-level vancomycin-resistant enterococci (VRE) in the 1980s (132, 133). VRE strains carried plasmids containing gene clusters, such as *vanA,* that could be transferred into *S. aureus*. These clusters encode enzymes that modify the C-terminal end of peptidoglycan precursors, usually from D-Ala-D-Ala to D-Ala-D-Lac, significantly reducing vancomycin’s binding affinity and thereby conferring resistance. Acquisition of this vancomycin resistance mechanism by MRSA would be catastrophic, as it would leave patients with no effective treatment options. The first VRSA strain was identified in 2002 in a patient in the United States (134). However, surprisingly, but fortunately, VRSA strains did not, so far, become dominant in hospital settings. This is possibly because the mechanism of vancomycin resistance in VRSA is incompatible with the mechanism of methicillin resistance in MRSA. Unlike native PBPs, PBP2A, the key determinant of MRSA resistance, probably cannot efficiently use the modified peptidoglycan precursor produced by VRSA in the presence of vancomycin (135). As a result, *S. aureus* strains, containing both *mecA* and *vanA*, can grow in the presence of high concentrations of either β-lactams or vancomycin, but not in the presence of high concentrations of both antibiotics (135), making this an interesting example of incompatibility of resistance mechanisms.

The lipopeptide antibiotic daptomycin represents a drug of last resort against MRSA and VISA infections. It blocks cell wall biosynthesis by a unique mechanism, targeting lipid-linked peptidoglycan precursors and inhibiting peptidoglycan synthesis, which leads to delocalization of the peptidoglycan synthesis machinery and massive membrane rearrangements (136). Daptomycin is also an important antibiotic for the treatment of vancomycin-resistant *E. faecium* or *E. faecalis* and of other Gram-positive pathogens. The mechanisms of daptomycin resistance, which can arise rapidly during therapy, were extensively explored in *S. aureus* to instruct related research in other bacteria (137). A variety of changes in the bacterial cell envelope contribute to daptomycin tolerance or resistance, including upregulation of the *dlt* operon, which is responsible for D-alanylation of teichoic acids and therefore increases the positive charge of the envelope (138). However, mutations in the membrane lipid synthase and translocase MprF represent the most consistently reported reason for resistance (139). MprF mutations leading to daptomycin resistance appear to alter the translocase activity of MprF, potentially leading to the expulsion of daptomycin or related lipopeptides from the bacterial membrane (140). Interestingly, daptomycin resistance was reported to sensitize MRSA to β-lactams, a phenomenon described as the “seesaw effect” (141–143). One explanation for this effect is that *mprF* mutations result in impaired function of PrsA, a chaperone required for the folding of PBPs, and consequently in reduced levels of β-lactam-induced PBP2A (141).

MprF was later identified in many other bacteria from virtually any taxa and even in Archaea. The *S. aureus* MprF represents the paradigm of a prokaryotic aminoacyl-phosphatidylglycerol synthase and transferase (144).

## *S. AUREUS* AS A MODEL FOR PATHOGENICITY

*S. aureus* has an aggressive virulence potential that is unique among bacterial pathogens, making it one of the most versatile and deadly causes of infections (127). Accordingly, its virulence factors, including adhesins, toxins, and immune evasins, were extensively studied, establishing *S. aureus* as a model pathogen for the study of invasive infections (145). Several of the virulence-associated factors are redundant, and many function only in a human host context, which remains a challenge for their characterization and for the identification of the most promising vaccine or anti-virulence therapy targets (127, 146). The invasion of sterile tissues by *S. aureus* is facilitated by adhesin proteins at the bacterial surface, which enable *S. aureus* to attach to host cells and matrix proteins (147). These surface proteins bind to a variety of host molecules, including cytokeratin, desmoglein, fibrinogen, fibronectin, loricrin, glycosidic structures of corneocytes, epithelial and endothelial cells, or serum proteins such as IgG. Many of these surface proteins are covalently tethered to the bacterial peptidoglycan by the sortase enzyme SrtA, through a specific pathway that involves transpeptidation of the protein at an LPxTG motif. The enzyme and pathway were first discovered in *S. aureus* by Olaf Schneewind (148) and later found in many other Bacillota (149).

The capacity of “microbial surface components recognizing adhesive matrix molecules” (MSCRAMMs) to bind specific host molecules represents an active area of research in *S. aureus* and many other pathogens (147). One such MSCRAMM, immunoglobulin-binding protein A, is extensively used for the isolation of IgG (150). Many infections caused by *S. aureus* and CoNS are initiated by the bacterium’s capacity to attach to the surface of implanted biomaterials and form biofilms (150). The dynamics of biofilm formation, along with the contribution of extracellular glycopolymers, such as the polymeric intercellular adhesin (PIA) (151), proteins (152), and DNA released from disrupted bacterial cells (153), were extensively studied in staphylococci and became a paradigm for understanding biofilms formed by other microorganisms. PIA plays a crucial role in the accumulation of staphylococcal cells and the formation of a robust biofilm on both implant materials and host tissues. The PIA biosynthesis operon (*ica*, intercellular adhesion) was first identified by Heilmann et al. (154). It is composed of the *icaADBC* biosynthetic genes and *icaR*, which encodes a repressor (155). Staphylococcal biofilm formation can also involve protein-based mechanisms as exemplified by the intercellular aggregation of *S. epidermidis* cells mediated by the surface protein Aap or the aggregation of *S. aureus* by the Aap ortholog SasG. Interestingly, Aap and SasG also contribute to bacterial adherence to human desquamated nasal epithelial cells and corneocytes, highlighting their dual function in colonization and infection (156).

The aggressive behavior of *S. aureus* in infections is largely due to its extensive toxin armory (157). This includes small membrane-damaging phenol-soluble modulin (PSM) peptides, such as *S.* aureus δ-toxin (158), large pore-forming toxins (159), superantigens (160), and several other toxic molecules. The various PSM peptides share several pathogenicity-related functions, such as the release of biofilm-associated bacterial cells, the mobilization of membrane vesicles containing proinflammatory molecules, the attraction of phagocytes, and, at high concentrations, the disruption of host cells (158, 161, 162). While *S. aureus* is a particularly strong PSM producer, most of the CoNS also produce such peptides. *S. aureus* also produces a variety of larger pore-forming toxins that oligomerize to form heptameric pores in host cell membranes (159). These toxins can consist of a single protein, such as α-toxin (Hla) (163), or two separate subunits, as seen in Panton-Valentine leukocidin (PVL), γ-haemolysin AB (HlgAB), γ-haemolysin CB (HlgCB), and leukocidin ED (LukED) (164). The study of these *S. aureus* toxins, including their structure, receptor interaction, and host-cell damaging functions, provided fundamental insights into similar toxins in other pathogens.

The virulence of *S. aureus* also results from the particular capacity to activate the adaptive immune system in an unspecific, exuberant fashion. Several superantigen toxins, including the toxic shock-associated toxin and a variety of enterotoxins, lead to unspecific activation of T cells and resulting cytokine storms (165). Superantigen toxins are also produced by other bacterial pathogens, such as *Streptococcus pyogenes*, the study of which was leveraged by the knowledge generated for *S. aureus* superantigen toxins (166).

*S. aureus* has multiple strategies to interfere with immune activation at various levels. Small secreted proteins interfere with the activation of phagocyte receptors (for instance, the formyl-peptide receptor 1 by the “chemotaxis-inhibitory protein of *S. aureus*” [CHIPS]) (167), the complement system (for instance, the C3 convertase by the “*Staphylococcus* complement inhibitor” [SCIN]) (168), or the oxidative burst-based antimicrobial mechanisms of phagocytes (for instance, the neutrophil myeloperoxidase by the “staphylococcal peroxidase inhibitor” [SPIN]) (169). While most of these proteins are not found in other pathogens, their discovery helped to elucidate crucial host defense mechanisms.

Another immune escape mechanism is based on the peptidoglycan O-acetylation by the OatA enzyme (85). Degradation of bacterial peptidoglycan by lysozyme in macrophage and dendritic cell phagosomes leads to the activation of the NLRP3 inflammasome, a cytosolic complex that regulates processing and secretion of interleukin IL-1β and IL-18. IL-1β is a key mediator of the inflammatory response and is essential for the host response and resistance to pathogens. Underhill and colleagues showed that the lysozyme-resistant *S. aureus* produced much less IL-1β (immune evasion) than the lysozyme-sensitive *oatA* mutant (170). It was assumed that lysozyme digestion of the *oatA* mutant multiplies the ligands that stimulate NLRP3 inflammasome. Particulate cell wall peptidoglycan from other skin commensals has the capacity to augment *S. aureus* pathogenesis strongly, albeit in an inflammasome-independent fashion (171).

*S. aureus* also evades recognition by the adaptive immune system, for instance, by varying the structure of the dominant surface antigen wall teichoic acid (WTA) (172). Altered glycosylation can be found in MRSA clones harboring a prophage-encoded accessory WTA glycosyl transferase (TarP) (173). Since WTA or related glycopolymers are found in all Bacillota, variation of their glycosylation patterns is likely to contribute to immune evasion in other Gram-positive pathogens.

*S. aureus* produces its virulence factors at different stages and in different types of infections, which requires sophisticated regulatory mechanisms. Among the multitude of its regulatory systems, the *agr* quorum-sensing system plays a central role (174). This system relies on the secretion of a modified peptide pheromone that accumulates in certain environments, triggering a two-component regulatory system that governs the expression of many key virulence factors (175). Since *agr*-related regulatory mechanisms have also been identified in several other bacterial pathogens, *S. aureus* became a model organism for exploring quorum-sensing mechanisms in virulence regulation (176).

## *S. AUREUS* AS A MODEL FOR INTERACTIONS BETWEEN MEMBERS OF THE MICROBIOME

**Fig 3:**
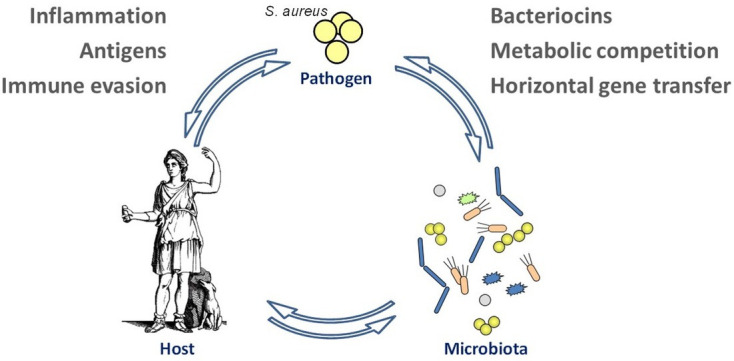
*S. aureus* interaction with host and microbiome. Topics for which *S. aureus* represents an important model organism are indicated on the upper left and right.

Although *S. aureus* is best known as an aggressive pathogen, it can switch between commensal and pathogenic lifestyles and is therefore a facultative pathogen (Fig. 3) (177). While the virulence mechanisms of *S. aureus* have been extensively studied, the processes of *S. aureus* colonization of the nasal microbiome and its interaction with other microbiome members and the human host have only been addressed recently. Notably, all ESKAPE pathogens alternate between commensal and pathogenic behavior, and *S. aureus* can be regarded as a model pathogen for the study of microbiome interaction and transition between different lifestyles (178). While intestinal microbiome science is a flourishing field of research, the microbiomes of the upper respiratory tract, the major habitat of *S. aureus*, are addressed by only a small number of laboratories. Such studies are particularly important because it became clear that humans who are not *S. aureus* carriers have a favorable composition of the nasal microbiome that precludes the establishment of *S. aureus* (179). Several mechanisms used by beneficial members of the nasal microbiome were identified, ranging from competition for essential nutrients (180) and deprivation of iron-binding siderophores (181) to the production of bacteriocin-like antimicrobials such as lugdunin and epifadin (182, 183). *S. aureus* is also found in low numbers in the intestine, which is increasingly considered as an alternative habitat (184). The development of *S. aureus*-excluding probiotic bacterial strains could become a sustainable way to protect at-risk patients from *S. aureus* infections (185, 186). Such approaches may motivate similar efforts against other ESKAPE pathogens (187).

*S. aureus* strains exchange genetic material, including genes encoding for resistance, fitness, and virulence factors, in the microbiome context, either with other *S. aureus* strains or even with other bacterial species, which strongly contributes to the evolution and adaptation of new clonal lineages (188). Transducing bacteriophages play a major role in such horizontal gene transfer events. They bind to target bacteria and discriminate between different host species by attaching to the species-specific structures of wall teichoic acid (WTA) glycopolymers at the staphylococcal surface (189). *S. aureus* became a model for the biosynthesis and function of WTA structure variation in the interaction with phages and host factors (172). Many of the exchanged genes are encoded on staphylococcal pathogenicity islands (SaPIs), highly mobile 15 kb genomic islands that hijack certain phages to facilitate their own horizontal transfer (190). Regulatory SaPI proteins sense the presence of such helper phages to induce the SaPI replication cycle, resulting in encapsidation of SaPI DNA in phage-like particles, enabling high-frequency transfer. SaPIs became paradigm elements for the study of phage-inducible chromosomal islands (PICIs), which are found in multiple bacterial species (191).

## FUTURE QUESTIONS

*S. aureus* will remain an important model for studying fundamental bacterial cellular processes, as well as microbe–microbe and microbe–host interactions. The growing threat of antibiotic-resistant bacterial infections, combined with the termination of antibiotic development programs by most pharmaceutical companies, underscores the urgency for more detailed studies on *S. aureus* antibiotic resistance, novel antimicrobial compounds, and new potential antibacterial targets. This should be informed by studies on cellular processes essential for growth and viability of bacterial cells. With no highly effective therapies for MRSA in sight, innovative strategies for preventing *S. aureus* infections are crucial. Understanding and harnessing the *S. aureus*-excluding capacities of nasal commensals represent a particularly promising field for future research. Furthermore, the development of an urgently needed vaccine against opportunistic *S. aureus* infections, which requires elucidation of protective antigens and of *S. aureus* immune evasion strategies, would represent a large step forward.
